# Aqua­(4-bromo­benzoato-κ*O*)bis­(1,10-phenanthroline-κ^2^ *N*,*N*′)zinc(II) 4-bromo­benzoate 1.5-hydrate

**DOI:** 10.1107/S160053681003864X

**Published:** 2010-10-02

**Authors:** Bi-Song Zhang, Su-Fang Ye, Yun-Xia Li, Wei Xu

**Affiliations:** a College of Materials Science and Chemical Engineering, Jinhua College of Profession and Technology, Jinhua, Zhejiang 321017, People’s Republic of China; b Municipal Key Laboratory of Inorganic Materials Chemistry, Institute for Solid State Chemistry, Ninbo University, Ningbo 315211, People’s Republic of China

## Abstract

In the title compound, [Zn(C_7_H_4_BrO_2_)(C_12_H_8_N_2_)_2_(H_2_O)](C_7_H_4_BrO_2_)·1.5H_2_O, the Zn^II^ atom is coordinated by four N atoms from two chelating 1,10-phenanthroline (phen) ligands, one O atom from a 4-bromo­benzoate ligand and one water mol­ecule, completing a distorted ZnN_4_O_2_ octa­hedral geometry. The two phen ligands exhibit nearly perfect coplanarity (r.m.s. deviations = 0.027 and 0.031 Å), making a dihedral angle of 85.7 (1)°. The mean inter­planar distances of 3.36 (2) and 3.41 (3) Å between adjacent phen ligands indicate π–π stacking inter­actions. The uncoordinated water mol­ecules are partly occupied. One carboxyl­ate O atom and two Br atoms are each disordered over two sites, with occupancy factors of 0.60 and 0.40. In the crystal structure, O—H⋯O and C—H⋯O hydrogen bonds and π–π stacking inter­actions link the complex cations, uncoordinated 4-bromo­benzoate anions and water mol­ecules into a three-dimensional supra­molecular network. An intra­molecular O—H⋯·O hydrogen bond is observed in the cation.

## Related literature

For related zinc(II) complexes with 1,10-phenanthroline ligand, see: Aghabozorg *et al.* (2005 ▶); Chen *et al.* (2006 ▶); Liu *et al.* (1998 ▶); Wei *et al.* (2002 ▶, 2004 ▶); Ye & Zhang (2010 ▶).
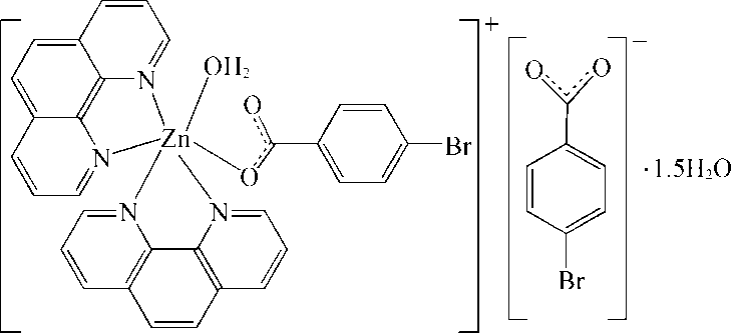


## Experimental

### 

#### Crystal data

[Zn(C_7_H_4_BrO_2_)(C_12_H_8_N_2_)_2_(H_2_O)](C_7_H_4_BrO_2_)·1.5H_2_O
*M* *_r_* = 870.84
Triclinic, 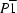

*a* = 10.170 (2) Å
*b* = 13.527 (3) Å
*c* = 15.908 (3) Å
α = 111.96 (3)°
β = 99.26 (3)°
γ = 102.25 (3)°
*V* = 1912.5 (10) Å^3^
*Z* = 2
Mo *K*α radiation
μ = 2.78 mm^−1^
*T* = 290 K
0.28 × 0.21 × 0.12 mm

#### Data collection

Rigaku R-AXIS RAPID diffractometer
Absorption correction: multi-scan (*ABSCOR*; Higashi, 1995 ▶) *T*_min_ = 0.500, *T*_max_ = 0.717
14823 measured reflections
6627 independent reflections
3645 reflections with *I* > 2σ(*I*)
*R*_int_ = 0.048

#### Refinement

*R*[*F* ^2^ > 2σ(*F* ^2^)] = 0.083
*wR*(*F* ^2^) = 0.307
*S* = 1.10
6627 reflections
497 parameters
6 restraints
H-atom parameters constrained
Δρ_max_ = 0.99 e Å^−3^
Δρ_min_ = −0.75 e Å^−3^

### 

Data collection: *RAPID-AUTO* (Rigaku, 1998 ▶); cell refinement: *RAPID-AUTO*; data reduction: *CrystalStructure* (Rigaku/MSC, 2002 ▶); program(s) used to solve structure: *SHELXS97* (Sheldrick, 2008 ▶); program(s) used to refine structure: *SHELXL97* (Sheldrick, 2008 ▶); molecular graphics: *SHELXTL* (Sheldrick, 2008 ▶); software used to prepare material for publication: *SHELXTL*.

## Supplementary Material

Crystal structure: contains datablocks I, global. DOI: 10.1107/S160053681003864X/hy2357sup1.cif

Structure factors: contains datablocks I. DOI: 10.1107/S160053681003864X/hy2357Isup2.hkl

Additional supplementary materials:  crystallographic information; 3D view; checkCIF report

## Figures and Tables

**Table 1:** Hydrogen-bond geometry (Å, °)

*D*—H⋯*A*	*D*—H	H⋯*A*	*D*⋯*A*	*D*—H⋯*A*
O5—H5*A*⋯O4^i^	0.82	1.88	2.676 (9)	163
O5—H5*B*⋯O2	0.82	1.87	2.639 (9)	155
O6—H6*A*⋯O2	0.82	1.87	2.672 (6)	168
O6—H6*B*⋯O3^i^	0.82	1.99	2.715 (5)	147
O6—H6*B*⋯O3′^i^	0.82	2.05	2.842 (5)	161
O7—H7*A*⋯O3^ii^	0.82	2.10	2.916 (11)	178
O7—H7*A*⋯O3′^ii^	0.82	1.71	2.513 (8)	168
O7—H7*B*⋯O6^iii^	0.82	2.01	2.823 (10)	172
O8—H8*A*⋯O6^iv^	0.82	2.08	2.808 (2)	148
O8—H8*B*⋯O9^v^	0.82	1.88	2.685 (2)	168
O9—H9*A*⋯O7	0.82	1.89	2.705 (6)	172
O9—H9*B*⋯O3^v^	0.82	2.08	2.876 (5)	164
C29—H29⋯O2^vi^	0.93	2.52	3.300 (14)	141

Symmetry codes: (i) 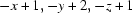; (ii) 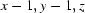; (iii) 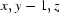; (iv) 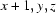; (v) 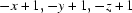; (vi) 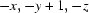.

**Fig. 1.**
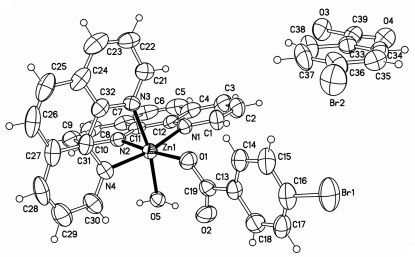
The molecular structure of the title compound. Displacement ellipsoids are drawn at the 50% probability level. The uncoordinated water molecules are omitted for clarity.

**Fig. 2.**
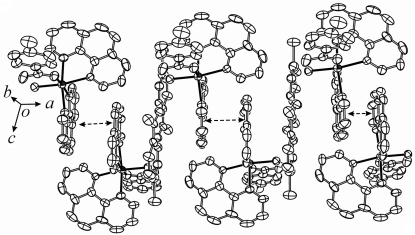
The π–π packing interactions (dashed double arrows), with a mean interplanar distance of 3.36 (2)Å.

## supplementary crystallographic information

### Comment

Zinc ion with 1,10-phenanthroline (phen) ligand can form tris(phen)zinc(II) (Aghabozorg *et al.*, 2005; Chen *et al.*, 2006; Liu *et al.*, 1998; Wei, Yuan *et al.*, 2004; Wei, Zheng *et al.*, 2002; Ye & Zhang, 2010). In this paper, we report the synthesis and structure of the title complex.

The title compound consists of [Zn(phen)_2_(C_7_H_4_BrO_2_)(H_2_O)]^+^ complex cations, 4-bromobenzoate anions and uncoordinated water molecules (Fig. 1). In the cation, the Zn^II^ atom is coordinated by four N atoms from two bidentate chelating phen ligands and one O atom from a 4-bromobenzoate anion and one water molecule, completing a distorted ZnN_4_O_2_ octahedral geometry. The Zn—N bond lengths are in the range of 2.137 (6)–2.217 (6)Å, and the Zn—O bond lengths are 2.101 (5) and 2.108 (5) Å. The two chelating phen ligands exhibit nearly perfect coplanarity, with a dihedral angle between the two phen ligands of 85.7 (1)°. The mean interplanar distances of 3.36 (2) and 3.41 (3)Å between adjacent phen ligands indicate π–π stacking interactions (Fig. 2). The complex cations, 4-bromobenzoate anions and uncoordinated water molecules are connected via π–π stacking interactions and O—H···O and C—H···O hydrogen bonds (Table 1) into a three-dimensional supramolecular network.

### Experimental

ZnCl_2_ (0.068 g, 0.50 mmol) was dissolved in appropriate amount of water, and then 1*M* Na_2_CO_3_ solution was added. ZnCO_3_ was obtained by filtration, which was then washed with distilled water for 5 times. The freshly prepared ZnCO_3_, 1,10-phenanthroline (phen.H_2_O) (0.049 g, 0.25 mmol) and 4-bromobenzoic acid (0.052 g, 0.25 mmol) in CH_3_OH/H_2_O (v/v = 1:2, 15 ml) were mixed and stirred for 2 h. Subsequently, the resulting cream suspension was heated in a 23 ml Teflon-lined stainless steel autoclave at 433 K for 5800 min. After the autoclave was cooled to room temperature in 2600 min, the solid was filtered off. The resulting filtrate was allowed to stand at room temperature, and slow evaporation for 2 months afforded colorless block single crystals.

### Refinement

C-bound H atoms were placed in calculated positions and refined using a riding model, with C—H = 0.93 Å and *U*_iso_(H) = 1.2*U*_eq_(C). The H atoms of water molecules were located in a difference Fourier map and refined as riding, with O—H distance restraints of 0.82 Å and *U*_iso_(H) = 1.5*U*_eq_(O). One carboxylate O atom (O3) and two Br atoms (Br1, Br2) are each disordered over two sites, with occupancy factors of 0.60 and 0.40. Two water molecules (O7, O9) are half-occupied and two water molecules (O6, O8) are quarter-occupied.

### Crystal data

**Table:**

[Zn(C_7_H_4_BrO_2_)(C_12_H_8_N_2_)_2_(H_2_O)](C_7_H_4_BrO_2_)·1.5H_2_O	*Z* = 2
*M**_r_* = 870.84	*F*(000) = 874
Triclinic, *P*1	*D*_x_ = 1.512 Mg m^−^^3^
Hall symbol: -P 1	Mo *K*α radiation, λ = 0.71073 Å
*a* = 10.170 (2) Å	Cell parameters from 9256 reflections
*b* = 13.527 (3) Å	θ = 3.1–25.0°
*c* = 15.908 (3) Å	µ = 2.78 mm^−^^1^
α = 111.96 (3)°	*T* = 290 K
β = 99.26 (3)°	Block, colorless
γ = 102.25 (3)°	0.28 × 0.21 × 0.12 mm
*V* = 1912.5 (10) Å^3^	

### Data collection

**Table:**

Rigaku R-AXIS RAPID diffractometer	6627 independent reflections
Radiation source: rotation anode	3645 reflections with *I* > 2σ(*I*)
graphite	*R*_int_ = 0.048
ω scans	θ_max_ = 25.0°, θ_min_ = 3.1°
Absorption correction: multi-scan (*ABSCOR*; Higashi, 1995)	*h* = −12→12
*T*_min_ = 0.500, *T*_max_ = 0.717	*k* = −14→16
14823 measured reflections	*l* = −18→18

### Refinement

**Table:**

Refinement on *F*^2^	Secondary atom site location: difference Fourier map
Least-squares matrix: full	Hydrogen site location: inferred from neighbouring sites
*R*[*F*^2^ > 2σ(*F*^2^)] = 0.083	H-atom parameters constrained
*wR*(*F*^2^) = 0.307	*w* = 1/[σ^2^(*F*_o_^2^) + (0.1855*P*)^2^] where *P* = (*F*_o_^2^ + 2*F*_c_^2^)/3
*S* = 1.10	(Δ/σ)_max_ = 0.001
6627 reflections	Δρ_max_ = 0.99 e Å^−^^3^
497 parameters	Δρ_min_ = −0.75 e Å^−^^3^
6 restraints	Extinction correction: *SHELXL97* (Sheldrick, 2008), Fc^*^=kFc[1+0.001xFc^2^λ^3^/sin(2θ)]^-1/4^
Primary atom site location: structure-invariant direct methods	Extinction coefficient: 0.020 (3)

### Fractional atomic coordinates and isotropic or equivalent isotropic displacement parameters (Å^2^)

**Table:**

	*x*	*y*	*z*	*U*_iso_*/*U*_eq_	Occ. (<1)
Zn1	0.32315 (9)	0.60478 (7)	0.26375 (6)	0.0578 (4)	
N1	0.4071 (7)	0.7042 (5)	0.4116 (4)	0.0631 (16)	
N2	0.3030 (6)	0.4787 (5)	0.3223 (5)	0.0645 (17)	
N3	0.5190 (7)	0.5826 (6)	0.2337 (4)	0.0618 (16)	
N4	0.2573 (8)	0.4811 (6)	0.1215 (5)	0.0688 (17)	
Br1	0.4506 (10)	1.1711 (9)	0.0925 (10)	0.129 (3)	0.60
Br2	0.8604 (8)	1.2210 (4)	0.0948 (4)	0.1240 (12)	0.60
O3	0.9222 (16)	1.1945 (19)	0.524 (2)	0.094 (5)	0.60
Br1'	0.3985 (18)	1.1522 (15)	0.0753 (15)	0.129 (3)	0.40
Br2'	0.8813 (14)	1.1856 (8)	0.0960 (7)	0.1240 (12)	0.40
O3'	0.983 (3)	1.217 (3)	0.531 (4)	0.094 (5)	0.40
O1	0.3511 (6)	0.7407 (5)	0.2275 (4)	0.0757 (16)	
O2	0.1453 (7)	0.7695 (6)	0.2255 (5)	0.095 (2)	
O4	0.9347 (7)	1.3775 (6)	0.5657 (5)	0.0898 (19)	
O5	0.1155 (5)	0.5971 (4)	0.2686 (4)	0.0648 (13)	
H5A	0.1016	0.5920	0.3164	0.097*	
H5B	0.1027	0.6509	0.2603	0.097*	
O6	0.047 (2)	0.9020 (12)	0.3554 (12)	0.056 (6)	0.25
H6A	0.0746	0.8549	0.3192	0.083*	0.25
H6B	0.0232	0.8740	0.3903	0.083*	0.25
O7	0.1680 (12)	0.1288 (10)	0.4825 (11)	0.101 (5)	0.50
H7A	0.0999	0.1490	0.4939	0.151*	0.50
H7B	0.1370	0.0614	0.4495	0.151*	0.50
O8	0.770 (2)	0.8729 (11)	0.2674 (12)	0.052 (5)	0.25
H8A	0.8534	0.8799	0.2726	0.078*	0.25
H8B	0.7531	0.9135	0.3148	0.078*	0.25
O9	0.2873 (13)	0.0195 (12)	0.5695 (12)	0.111 (5)	0.50
H9A	0.2514	0.0479	0.5382	0.166*	0.50
H9B	0.2409	−0.0465	0.5449	0.166*	0.50
C1	0.4586 (11)	0.8132 (7)	0.4504 (7)	0.088 (3)	
H1	0.4703	0.8514	0.4128	0.106*	
C2	0.4966 (13)	0.8729 (10)	0.5491 (8)	0.115 (4)	
H2	0.5355	0.9500	0.5768	0.137*	
C3	0.4754 (13)	0.8154 (11)	0.6053 (8)	0.111 (4)	
H3	0.4972	0.8542	0.6703	0.133*	
C4	0.4222 (10)	0.7012 (10)	0.5635 (6)	0.086 (3)	
C5	0.4006 (12)	0.6413 (14)	0.6147 (8)	0.110 (4)	
H5	0.4203	0.6775	0.6798	0.132*	
C6	0.3493 (12)	0.5263 (14)	0.5696 (9)	0.108 (4)	
H6	0.3351	0.4864	0.6057	0.130*	
C7	0.3171 (9)	0.4659 (10)	0.4699 (7)	0.080 (3)	
C8	0.2664 (11)	0.3491 (11)	0.4196 (10)	0.098 (4)	
H8	0.2520	0.3052	0.4523	0.118*	
C9	0.2384 (10)	0.2998 (9)	0.3258 (10)	0.098 (3)	
H9	0.2078	0.2225	0.2933	0.118*	
C10	0.2569 (8)	0.3694 (7)	0.2769 (7)	0.076 (2)	
H10	0.2359	0.3365	0.2116	0.091*	
C11	0.3352 (8)	0.5271 (7)	0.4181 (6)	0.063 (2)	
C12	0.3869 (8)	0.6451 (7)	0.4647 (6)	0.066 (2)	
C13	0.3092 (10)	0.8828 (6)	0.1863 (6)	0.067 (2)	
C14	0.4391 (11)	0.9167 (7)	0.1788 (6)	0.078 (2)	
H14	0.5045	0.8831	0.1938	0.094*	
C15	0.4792 (13)	1.0015 (8)	0.1490 (8)	0.099 (3)	
H15	0.5695	1.0243	0.1442	0.119*	
C16	0.3790 (14)	1.0504 (8)	0.1268 (7)	0.094 (3)	
C17	0.2486 (13)	1.0202 (8)	0.1351 (7)	0.088 (3)	
H17	0.1845	1.0551	0.1206	0.106*	
C18	0.2091 (11)	0.9364 (8)	0.1656 (7)	0.086 (3)	
H18	0.1195	0.9159	0.1723	0.103*	
C19	0.2657 (9)	0.7919 (7)	0.2155 (6)	0.065 (2)	
C21	0.6460 (9)	0.6319 (8)	0.2868 (7)	0.075 (2)	
H21	0.6594	0.6902	0.3450	0.090*	
C22	0.7646 (10)	0.6040 (10)	0.2633 (9)	0.088 (3)	
H22	0.8530	0.6421	0.3046	0.106*	
C23	0.7458 (13)	0.5192 (11)	0.1779 (10)	0.106 (4)	
H23	0.8219	0.4976	0.1606	0.128*	
C24	0.6103 (13)	0.4636 (9)	0.1153 (8)	0.087 (3)	
C25	0.5783 (17)	0.3763 (10)	0.0221 (10)	0.105 (4)	
H25	0.6503	0.3517	0.0005	0.126*	
C26	0.4516 (19)	0.3304 (9)	−0.0335 (8)	0.110 (4)	
H26	0.4370	0.2754	−0.0936	0.132*	
C27	0.3335 (14)	0.3622 (8)	−0.0046 (7)	0.089 (3)	
C28	0.1928 (17)	0.3188 (8)	−0.0591 (7)	0.106 (4)	
H28	0.1695	0.2654	−0.1208	0.128*	
C29	0.0939 (14)	0.3544 (10)	−0.0220 (8)	0.108 (3)	
H29	0.0013	0.3245	−0.0574	0.129*	
C30	0.1286 (11)	0.4347 (9)	0.0678 (6)	0.088 (3)	
H30	0.0577	0.4578	0.0923	0.106*	
C31	0.3609 (10)	0.4455 (6)	0.0874 (6)	0.068 (2)	
C32	0.5005 (9)	0.4985 (7)	0.1489 (6)	0.068 (2)	
C33	0.9135 (8)	1.2651 (7)	0.4085 (6)	0.068 (2)	
C34	0.9238 (9)	1.3530 (7)	0.3822 (6)	0.071 (2)	
H34	0.9400	1.4249	0.4279	0.085*	
C35	0.9104 (10)	1.3356 (9)	0.2890 (7)	0.082 (3)	
H35	0.9201	1.3956	0.2729	0.099*	
C36	0.8833 (11)	1.2317 (9)	0.2224 (8)	0.097 (3)	
C37	0.8712 (12)	1.1411 (8)	0.2434 (8)	0.107 (4)	
H37	0.8521	1.0700	0.1958	0.129*	
C38	0.8876 (11)	1.1564 (8)	0.3362 (7)	0.090 (3)	
H38	0.8818	1.0956	0.3512	0.107*	
C39	0.9305 (9)	1.2826 (8)	0.5071 (7)	0.075 (2)	

### Atomic displacement parameters (Å^2^)

**Table:**

	*U*^11^	*U*^22^	*U*^33^	*U*^12^	*U*^13^	*U*^23^
Zn1	0.0629 (6)	0.0564 (6)	0.0544 (6)	0.0179 (4)	0.0156 (4)	0.0236 (4)
N1	0.066 (4)	0.061 (4)	0.056 (4)	0.016 (3)	0.013 (3)	0.021 (3)
N2	0.056 (4)	0.064 (4)	0.075 (5)	0.018 (3)	0.014 (3)	0.032 (4)
N3	0.056 (4)	0.071 (4)	0.054 (4)	0.017 (3)	0.009 (3)	0.026 (3)
N4	0.075 (5)	0.064 (4)	0.060 (4)	0.010 (4)	0.013 (4)	0.026 (3)
Br1	0.205 (8)	0.078 (4)	0.117 (5)	0.024 (4)	0.062 (5)	0.060 (4)
Br2	0.174 (3)	0.123 (4)	0.0702 (9)	0.047 (2)	0.0305 (13)	0.035 (2)
O3	0.101 (15)	0.106 (11)	0.113 (8)	0.042 (13)	0.051 (16)	0.070 (10)
Br1'	0.205 (8)	0.078 (4)	0.117 (5)	0.024 (4)	0.062 (5)	0.060 (4)
Br2'	0.174 (3)	0.123 (4)	0.0702 (9)	0.047 (2)	0.0305 (13)	0.035 (2)
O3'	0.101 (15)	0.106 (11)	0.113 (8)	0.042 (13)	0.051 (16)	0.070 (10)
O1	0.082 (4)	0.074 (4)	0.084 (4)	0.028 (3)	0.021 (3)	0.044 (3)
O2	0.081 (4)	0.098 (5)	0.132 (6)	0.030 (4)	0.017 (4)	0.079 (5)
O4	0.104 (5)	0.104 (5)	0.076 (4)	0.040 (4)	0.040 (4)	0.042 (4)
O5	0.062 (3)	0.068 (3)	0.066 (4)	0.016 (3)	0.017 (3)	0.032 (3)
O6	0.128 (16)	0.034 (8)	0.059 (11)	0.056 (11)	0.079 (12)	0.038 (9)
O7	0.077 (8)	0.072 (8)	0.198 (16)	0.037 (7)	0.057 (9)	0.087 (10)
O8	0.082 (13)	0.011 (7)	0.039 (10)	0.005 (8)	−0.001 (9)	−0.003 (7)
O9	0.081 (9)	0.097 (10)	0.175 (16)	0.025 (8)	0.024 (9)	0.084 (11)
C1	0.106 (7)	0.056 (5)	0.085 (7)	0.017 (5)	0.020 (6)	0.018 (5)
C2	0.133 (10)	0.089 (8)	0.078 (8)	0.043 (7)	−0.003 (7)	−0.003 (6)
C3	0.128 (10)	0.114 (9)	0.068 (7)	0.064 (8)	0.010 (7)	0.004 (7)
C4	0.092 (7)	0.145 (10)	0.040 (5)	0.067 (7)	0.031 (5)	0.037 (6)
C5	0.094 (8)	0.181 (13)	0.073 (8)	0.064 (10)	0.034 (6)	0.056 (9)
C6	0.087 (7)	0.210 (14)	0.110 (10)	0.079 (9)	0.059 (7)	0.123 (11)
C7	0.069 (5)	0.125 (8)	0.097 (7)	0.045 (6)	0.045 (5)	0.083 (7)
C8	0.080 (7)	0.114 (9)	0.159 (12)	0.039 (7)	0.056 (8)	0.104 (9)
C9	0.068 (6)	0.081 (7)	0.162 (12)	0.020 (5)	0.031 (7)	0.071 (8)
C10	0.061 (5)	0.071 (6)	0.098 (7)	0.014 (4)	0.018 (5)	0.042 (5)
C11	0.050 (4)	0.087 (6)	0.071 (6)	0.029 (4)	0.022 (4)	0.047 (5)
C12	0.060 (5)	0.077 (5)	0.067 (5)	0.036 (4)	0.019 (4)	0.028 (5)
C13	0.093 (6)	0.055 (5)	0.055 (5)	0.019 (5)	0.014 (4)	0.028 (4)
C14	0.106 (7)	0.064 (5)	0.067 (6)	0.026 (5)	0.036 (5)	0.026 (4)
C15	0.133 (9)	0.063 (6)	0.104 (8)	0.023 (6)	0.058 (7)	0.030 (6)
C16	0.139 (10)	0.063 (6)	0.076 (7)	0.020 (7)	0.033 (7)	0.029 (5)
C17	0.137 (9)	0.064 (5)	0.066 (6)	0.034 (6)	0.014 (6)	0.034 (5)
C18	0.092 (7)	0.083 (6)	0.081 (7)	0.022 (6)	0.012 (5)	0.039 (5)
C19	0.067 (5)	0.066 (5)	0.052 (5)	0.013 (5)	0.007 (4)	0.023 (4)
C21	0.075 (6)	0.080 (6)	0.082 (6)	0.021 (5)	0.019 (5)	0.050 (5)
C22	0.062 (5)	0.113 (8)	0.123 (9)	0.034 (6)	0.036 (6)	0.075 (8)
C23	0.110 (9)	0.130 (10)	0.162 (13)	0.070 (9)	0.091 (9)	0.109 (10)
C24	0.118 (9)	0.098 (7)	0.109 (8)	0.066 (7)	0.075 (7)	0.076 (7)
C25	0.169 (12)	0.094 (8)	0.116 (10)	0.076 (9)	0.100 (10)	0.066 (8)
C26	0.214 (15)	0.064 (6)	0.077 (8)	0.059 (9)	0.076 (9)	0.033 (6)
C27	0.160 (10)	0.061 (5)	0.063 (6)	0.036 (7)	0.056 (7)	0.032 (5)
C28	0.187 (13)	0.056 (6)	0.048 (6)	0.007 (7)	0.014 (8)	0.015 (5)
C29	0.129 (10)	0.101 (8)	0.063 (7)	0.012 (8)	0.006 (7)	0.023 (6)
C30	0.098 (7)	0.093 (7)	0.054 (6)	0.012 (6)	0.010 (5)	0.025 (5)
C31	0.097 (6)	0.054 (4)	0.068 (6)	0.026 (5)	0.037 (5)	0.034 (4)
C32	0.084 (6)	0.068 (5)	0.085 (6)	0.033 (5)	0.039 (5)	0.054 (5)
C33	0.058 (5)	0.071 (5)	0.076 (6)	0.019 (4)	0.029 (4)	0.029 (5)
C34	0.073 (5)	0.065 (5)	0.074 (6)	0.022 (4)	0.027 (5)	0.025 (4)
C35	0.084 (6)	0.094 (7)	0.083 (7)	0.032 (6)	0.028 (5)	0.047 (6)
C36	0.086 (7)	0.084 (7)	0.094 (8)	−0.003 (6)	0.023 (6)	0.023 (6)
C37	0.121 (9)	0.061 (6)	0.097 (9)	0.000 (6)	0.039 (7)	−0.002 (6)
C38	0.109 (8)	0.063 (5)	0.085 (7)	0.009 (5)	0.035 (6)	0.024 (5)
C39	0.083 (6)	0.083 (6)	0.079 (6)	0.039 (5)	0.044 (5)	0.040 (5)

### Geometric parameters (Å, °)

**Table:**

Zn1—O1	2.101 (5)	C8—H8	0.9300
Zn1—O5	2.108 (5)	C9—C10	1.429 (13)
Zn1—N1	2.137 (6)	C9—H9	0.9300
Zn1—N4	2.138 (7)	C10—H10	0.9300
Zn1—N3	2.177 (6)	C11—C12	1.416 (12)
Zn1—N2	2.217 (6)	C13—C14	1.343 (13)
N1—C1	1.310 (11)	C13—C18	1.433 (12)
N1—C12	1.374 (10)	C13—C19	1.479 (11)
N2—C10	1.314 (11)	C14—C15	1.406 (13)
N2—C11	1.365 (10)	C14—H14	0.9300
N3—C21	1.299 (10)	C15—C16	1.394 (15)
N3—C32	1.352 (10)	C15—H15	0.9300
N4—C30	1.314 (11)	C16—C17	1.345 (15)
N4—C31	1.357 (10)	C17—C18	1.401 (12)
Br1—C16	1.946 (15)	C17—H17	0.9300
Br2—C36	1.952 (13)	C18—H18	0.9300
O3—C39	1.301 (14)	C21—C22	1.404 (12)
Br1'—C16	1.84 (2)	C21—H21	0.9300
Br2'—C36	1.864 (15)	C22—C23	1.362 (16)
O3'—C39	1.266 (18)	C22—H22	0.9300
O1—C19	1.254 (9)	C23—C24	1.419 (16)
O2—C19	1.248 (10)	C23—H23	0.9300
O4—C39	1.260 (10)	C24—C32	1.408 (12)
O5—H5A	0.8200	C24—C25	1.439 (16)
O5—H5B	0.8200	C25—C26	1.307 (16)
O6—H6A	0.8200	C25—H25	0.9300
O6—H6B	0.8200	C26—C27	1.445 (17)
O7—H7A	0.8200	C26—H26	0.9300
O7—H7B	0.8200	C27—C31	1.411 (12)
O8—H8A	0.8200	C27—C28	1.416 (16)
O8—H8B	0.8200	C28—C29	1.332 (17)
O9—H9A	0.8200	C28—H28	0.9300
O9—H9B	0.8200	C29—C30	1.364 (14)
C1—C2	1.411 (14)	C29—H29	0.9300
C1—H1	0.9300	C30—H30	0.9300
C2—C3	1.401 (17)	C31—C32	1.444 (13)
C2—H2	0.9300	C33—C34	1.389 (11)
C3—C4	1.371 (16)	C33—C38	1.424 (12)
C3—H3	0.9300	C33—C39	1.472 (12)
C4—C5	1.358 (16)	C34—C35	1.390 (12)
C4—C12	1.410 (11)	C34—H34	0.9300
C5—C6	1.381 (18)	C35—C36	1.342 (14)
C5—H5	0.9300	C35—H35	0.9300
C6—C7	1.428 (16)	C36—C37	1.372 (15)
C6—H6	0.9300	C37—C38	1.389 (13)
C7—C11	1.375 (11)	C37—H37	0.9300
C7—C8	1.406 (15)	C38—H38	0.9300
C8—C9	1.338 (15)		
			
O1—Zn1—O5	93.7 (2)	C16—C15—H15	121.1
O1—Zn1—N1	95.2 (2)	C14—C15—H15	121.1
O5—Zn1—N1	93.8 (2)	C17—C16—C15	121.9 (9)
O1—Zn1—N4	94.6 (2)	C17—C16—Br1'	111.3 (10)
O5—Zn1—N4	91.9 (2)	C15—C16—Br1'	126.6 (11)
N1—Zn1—N4	168.3 (2)	C17—C16—Br1	124.4 (9)
O1—Zn1—N3	89.9 (2)	C15—C16—Br1	113.5 (10)
O5—Zn1—N3	168.8 (2)	C16—C17—C18	120.2 (9)
N1—Zn1—N3	96.5 (2)	C16—C17—H17	119.9
N4—Zn1—N3	77.1 (3)	C18—C17—H17	119.9
O1—Zn1—N2	172.2 (2)	C17—C18—C13	118.8 (10)
O5—Zn1—N2	86.1 (2)	C17—C18—H18	120.6
N1—Zn1—N2	77.0 (3)	C13—C18—H18	120.6
N4—Zn1—N2	93.2 (3)	O2—C19—O1	123.8 (8)
N3—Zn1—N2	91.8 (2)	O2—C19—C13	118.0 (8)
C1—N1—C12	121.6 (8)	O1—C19—C13	118.2 (8)
C1—N1—Zn1	124.2 (6)	N3—C21—C22	125.1 (10)
C12—N1—Zn1	113.7 (5)	N3—C21—H21	117.4
C10—N2—C11	119.4 (8)	C22—C21—H21	117.4
C10—N2—Zn1	128.5 (6)	C23—C22—C21	117.7 (10)
C11—N2—Zn1	111.9 (5)	C23—C22—H22	121.2
C21—N3—C32	117.1 (7)	C21—C22—H22	121.2
C21—N3—Zn1	129.8 (6)	C22—C23—C24	120.2 (9)
C32—N3—Zn1	112.9 (5)	C22—C23—H23	119.9
C30—N4—C31	118.7 (8)	C24—C23—H23	119.9
C30—N4—Zn1	126.5 (6)	C32—C24—C23	116.0 (10)
C31—N4—Zn1	114.7 (6)	C32—C24—C25	118.7 (12)
C19—O1—Zn1	128.5 (6)	C23—C24—C25	125.2 (10)
Zn1—O5—H5A	111.0	C26—C25—C24	122.4 (11)
Zn1—O5—H5B	104.8	C26—C25—H25	118.8
H5A—O5—H5B	117.6	C24—C25—H25	118.8
H6A—O6—H6B	103.1	C25—C26—C27	122.2 (11)
H7A—O7—H7B	105.2	C25—C26—H26	118.9
H8A—O8—H8B	113.9	C27—C26—H26	118.9
H9A—O9—H9B	105.3	C31—C27—C28	116.4 (10)
N1—C1—C2	120.1 (10)	C31—C27—C26	116.7 (12)
N1—C1—H1	119.9	C28—C27—C26	126.9 (11)
C2—C1—H1	119.9	C29—C28—C27	120.2 (10)
C3—C2—C1	119.7 (11)	C29—C28—H28	119.9
C3—C2—H2	120.1	C27—C28—H28	119.9
C1—C2—H2	120.1	C28—C29—C30	119.9 (12)
C4—C3—C2	119.3 (10)	C28—C29—H29	120.0
C4—C3—H3	120.3	C30—C29—H29	120.0
C2—C3—H3	120.3	N4—C30—C29	123.3 (11)
C5—C4—C3	121.7 (11)	N4—C30—H30	118.4
C5—C4—C12	119.3 (12)	C29—C30—H30	118.4
C3—C4—C12	118.9 (10)	N4—C31—C27	121.4 (9)
C4—C5—C6	119.7 (11)	N4—C31—C32	116.8 (7)
C4—C5—H5	120.2	C27—C31—C32	121.7 (9)
C6—C5—H5	120.2	N3—C32—C24	123.8 (9)
C5—C6—C7	122.7 (10)	N3—C32—C31	118.0 (7)
C5—C6—H6	118.6	C24—C32—C31	118.1 (9)
C7—C6—H6	118.6	C34—C33—C38	117.6 (8)
C11—C7—C8	116.9 (10)	C34—C33—C39	121.9 (8)
C11—C7—C6	117.4 (11)	C38—C33—C39	120.5 (8)
C8—C7—C6	125.7 (10)	C33—C34—C35	121.2 (8)
C9—C8—C7	121.5 (9)	C33—C34—H34	119.4
C9—C8—H8	119.2	C35—C34—H34	119.4
C7—C8—H8	119.2	C36—C35—C34	119.7 (10)
C8—C9—C10	118.2 (10)	C36—C35—H35	120.2
C8—C9—H9	120.9	C34—C35—H35	120.2
C10—C9—H9	120.9	C35—C36—C37	122.0 (11)
N2—C10—C9	121.4 (10)	C35—C36—Br2'	129.0 (9)
N2—C10—H10	119.3	C37—C36—Br2'	108.4 (9)
C9—C10—H10	119.3	C35—C36—Br2	114.5 (9)
N2—C11—C7	122.5 (9)	C37—C36—Br2	123.4 (9)
N2—C11—C12	117.8 (7)	C36—C37—C38	119.6 (9)
C7—C11—C12	119.7 (9)	C36—C37—H37	120.2
N1—C12—C4	120.2 (9)	C38—C37—H37	120.2
N1—C12—C11	118.6 (7)	C37—C38—C33	119.9 (9)
C4—C12—C11	121.2 (9)	C37—C38—H38	120.1
C14—C13—C18	119.1 (8)	C33—C38—H38	120.1
C14—C13—C19	122.2 (8)	O4—C39—O3'	122 (3)
C18—C13—C19	118.7 (8)	O4—C39—O3	125.6 (16)
C13—C14—C15	122.0 (9)	O4—C39—C33	117.3 (7)
C13—C14—H14	119.0	O3'—C39—C33	117 (3)
C15—C14—H14	119.0	O3—C39—C33	116.5 (16)
C16—C15—C14	117.8 (10)		

### Hydrogen-bond geometry (Å, °)

**Table:**

*D*—H···*A*	*D*—H	H···*A*	*D*···*A*	*D*—H···*A*
O5—H5A···O4^i^	0.82	1.88	2.676 (9)	163
O5—H5B···O2	0.82	1.87	2.639 (9)	155
O6—H6A···O2	0.82	1.87	2.672 (6)	168
O6—H6B···O3^i^	0.82	1.99	2.715 (5)	147
O6—H6B···O3'^i^	0.82	2.05	2.842 (5)	161
O7—H7A···O3^ii^	0.82	2.10	2.916 (11)	178
O7—H7A···O3'^ii^	0.82	1.71	2.513 (8)	168
O7—H7B···O6^iii^	0.82	2.01	2.823 (10)	172
O8—H8A···O6^iv^	0.82	2.08	2.808 (2)	148
O8—H8B···O9^v^	0.82	1.88	2.685 (2)	168
O9—H9A···O7	0.82	1.89	2.705 (6)	172
O9—H9B···O3^v^	0.82	2.08	2.876 (5)	164
C29—H29···O2^vi^	0.93	2.52	3.300 (14)	141

Symmetry codes: (i) −*x*+1, −*y*+2, −*z*+1;  (ii) *x*−1, *y*−1, *z*; (iii) *x*, *y*−1, *z*; (iv) *x*+1, *y*, *z*; (v) −*x*+1, −*y*+1, −*z*+1; (vi) −*x*, −*y*+1, −*z*.
